# Single-Cell Analysis of Target Antigens of CAR-T Reveals a Potential Landscape of “On-Target, Off-Tumor Toxicity”

**DOI:** 10.3389/fimmu.2021.799206

**Published:** 2021-12-16

**Authors:** Yinyin Zhang, Yingmei Li, Weijie Cao, Fang Wang, Xinsheng Xie, Yadan Li, Xiaoyi Wang, Rong Guo, Zhongxing Jiang, Rongqun Guo

**Affiliations:** ^1^ Department of Hematology, The First Affiliated Hospital of Zhengzhou University, Zhengzhou, China; ^2^ The Affiliated Cancer Hospital of Zhengzhou University, Henan Cancer Hospital, Zhengzhou, China; ^3^ Department of Pediatric Hematology and Oncology, The First Affiliated Hospital of Zhengzhou University, Zhengzhou, China

**Keywords:** single-cell RNA sequencing, chimeric antigen receptor T cells (CAR T cells), target antigen, on-target, off-tumor toxicity, malignant cells

## Abstract

Cellular immunotherapy represented by CD19-directed chimeric antigen receptor T (CAR-T) cells has achieved great success in recent years. An increasing number of CAR-T therapies are being developed for cancer treatment, but the frequent and varied adverse events, such as “on-target, off-tumor toxicity”, limit CAR-T application. Here, we identify the target antigen expression patterns of CAR therapies in 18 tissues and organs (peripheral blood mononuclear cells, bone marrow, lymph nodes, spleen, heart, ascending aortic tissue, trachea, lung, skin, kidney, bladder, esophagus, stomach, small intestine, rectum, liver, common bile duct, and pancreas) from healthy human samples. The atlas determines target antigens expressed on some normal cell types, which facilitates elucidating the cause of “on-target, off-tumor toxicity” in special tissues and organs by targeting some antigens, but not others. Moreover, we describe the target antigen expression patterns of B-lineage-derived malignant cells, acute myeloid leukemia (AML), and solid tumors. Overall, the present study indicates the pathogenesis of “on-target, off-tumor toxicity” during CAR therapies and provides guidance on taking preventive measures during CAR treatment.

## Introduction

The clinical applications of CD19-directed chimeric antigen receptor T (CAR-T) cell therapies have brought about a comprehensive innovation in the field of tumor treatment (1). Encouraged by this, researchers are trying to extend the applications of CAR-T to other hematological tumors while making a breakthrough in CAR-T treatment of solid tumors. Compared with conventional chemotherapy and radiotherapy, CAR-T therapy has the advantage of higher targeting specificity.

However, target antigens with universal practical value are usually also expressed in normal cells, such as CD19 in the normal B-cell lineage. Therefore, CAR-T has a significant defect, namely “on-target, off-tumor toxicity”. The following evidence shows that the “on-target, off-tumor toxicity” of CAR-T treatment is widespread, although a large part of others have not been identified or overlaps with other symptoms. CD19-targeting CAR-T therapy not only leads to B lymphocytopenia but also is related to neurotoxicity. A unique study showed that the occurrence of neurotoxicity during CD19-directed CAR-T treatment may be relevant to CD19-expressing brain mural cells (2). CD38-directed CAR-T cells have shown strong anti-multiple myeloma (MM) effect, and its “on-target off-tumor effect” against normal hematopoietic cells has been identified (3). CD123 (IL3RA)-directed CAR-T cells attack AML cells but inhibit normal hematopoiesis (4). Furthermore, the “on-target, off-tumor toxicity” effect of CAR-T therapy is significant in treating solid tumor types. For example, HER2-directed CAR-T cells may cause respiratory distress and cardiac arrest owing to the location of HER2-expressing normal cell types (5). In addition, carbonic anhydrase IX (CAIX)-directed CAR-T and fibroblast activation protein-α (FAP)-directed CAR-T treatment exhibit observable “on-target, off-tumor toxicity” (6, 7). Indeed, some “on-target, off-tumor effects” were not foreseen in preclinical animal experiments because of variability in cross-species reactivity to nonhuman target antigens, which usually provide a false sense of safety (8).

From the aspect of single-cell RNA-seq (scRNA-seq) in this study, we analyzed 121 target antigen expression patterns of CAR-T in 18 tissues and organs derived from normal human samples. Next, we analyzed the CAR safety of targeting B lineage-related target antigens (CD19, MS4A1, CD22, TNFRSF17, CD38, SLAMF7, and TNFRSF8), AML-related target antigens (CD33, CD123, and CLEC12A), and solid tumor-related target antigens (GPC3, B4GALNT1, and ERBB2). Finally, we compared the expression levels of antigens in malignant cells and nonmalignant cells. Broadly, our study highlights the diversity of potential “on-target, off-tumor effects” of CAR-T treatment. It is also indicated that increasingly enriched single cell omics datasets are becoming powerful tools to guide medical practice.

## Results

### A Single-Cell Atlas of the “On-Target, Off-Tumor Toxicities” of CAR-T in Tissues and Organs From Healthy Human Samples

**Figure 1:**
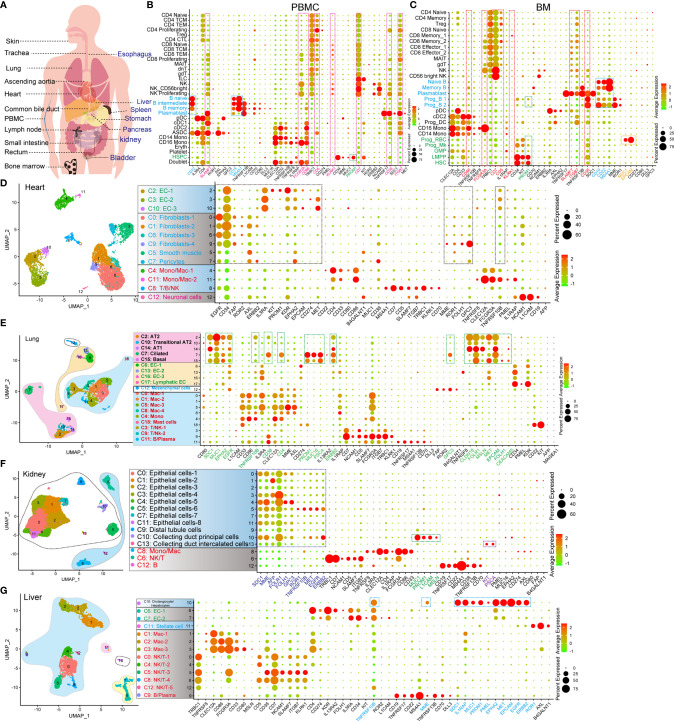
Identification of the expression pattern of CAR target antigens in normal tissues and organs at single-cell transcriptome level. **(A)** Schematic representation of selected tissues and organs for analyzing target antigens. **(B)** Dot plot shows the expression levels of CAR target antigens in PBMCs. **(C)** Dot plot shows the expression levels of CAR target antigens in BM. UMAP projections of heart-derived cells **(D)**, lung-derived cells **(E)**, kidney-derived cells **(F)**, and liver-derived cells **(G)**, colored by clusters, and dot plots showing the expression level of CAR target antigens in different clusters.

The expression of CAR-target antigens in normal organs and tissues leads to the risk of “on-target, off-tumor” targeting (9). Accordingly, we examined the expression pattern of CAR-target antigens in normal cell types at the scRNA-seq level (**Figure 1A** and **Table 1**), which has not yet been characterized. We first examined the antigen expression pattern in hematopoietic and immune systems (peripheral blood mononuclear cells [PBMCs], bone marrow [BM], lymph node [LN], and spleen [SP]), and found some antigens (such as ITGB7, CD38, CD7, KLRK1, CD5, CD86, FCGR3A, and SLAMF7) are expressed in multiple immune and hematopoietic lineages (**Figures 1B, C** and **Supplementary Figures 1A**, **2C, D**). B lineage-specific antigens CD19, MS4A1 (CD20), and CD22 are highly expressed in B progenitors (Prog B), mature B cells, and plasmablasts, which were identified at the mRNA and/or protein levels (**Supplementary Figures 2A, B**). As previously reported (10), we found that PROM1 (CD133) is strongly expressed in hematopoietic stem/progenitor cells (HSPCs), such as hematopoietic stem cells (HSCs), lymphoid-primed multipotent progenitors (LMPP), megakaryocyte progenitors (Prog Mk), and Prog B (**Figure 1C**), indicating that CD133 taken as a target may trigger the “on-target, off-tumor” myeloablative toxicity. Significantly, EPCAM or MUC1-directed CAR-T therapies may trigger strong cytotoxicity against erythroid progenitors (Prog RBC) in BM (**Figure 1C**).

**Table 1:** Common CAR-target antigens (57 genes).

CAR-target Antigens (57 genes)	CD19, MS4A1, CD22, TNFRSF17, CD38, SDC1, TNFRSF8, IL3RA, CD7, NCAM1, CD34, CLEC12A, CD4, MME, CD5, SLAMF7, IL1RAP, FCGR3A, ITGB7, TNFRSF13B, TRBC1, CD33, ROR1, MUC1, KLRK1, KIT, CD274, CD70, PROM1, AFP, AXL, CD80, CD86, DLL3, TNFRSF10B, FAP, MAGEA1, MAGEA4, MUC16, PMEL, ROR2, KDR, EPHA2, L1CAM, CLDN18, PSCA, FOLR1, IL13RA2, MET, EPCAM, EGFR, FOLH1, GPC3, CEACAM5, ERBB2, B4GALNT1, MSLN

Subsequently, the expression patterns of CAR-T target antigens in non-immune tissues and organs were examined. EGFR, CD34, FAP, ROR2, AXL, ERBB2, IL3RA, KIT, PROM1, KDR, EPHA2, CD274, MET, MME, ROR1, FOLH1, GPC3, and TNFRSF10B are highly expressed in cardiac endothelial cells (ECs), fibroblasts, and smooth muscle cells (SMCs)/pericytes (**Figure 1D** and **Supplementary Figure 1B**). Fibroblasts and SMCs of ascending aortic tissue express PROM1, ROR1, IL13RA2, ERBB2, FAP, CD34, AXL, EGFR, and GPC3 at high levels (**Supplementary Figure 2E**). Mast cells of ascending aortic tissue highly express CD22, which may result in a mast cell-related immune reaction when CD22 is used as a target. The epithelial components (alveolar type 1 cells [AT1], alveolar type 2 cells [AT2], ciliated cells, and basal cells) of the lung strongly express multiple target antigens, such as SDC1, MUC1, EPHA2, EGFR, TNFRSF10B, CD38, CD4, PROM1, MUC16, PSCA, ERBB2, GPC3, CLDN18, FOLR1, MSLN, MET, EPCAM, FOLH1, and CEACAM5 (**Figure 1E**). When these target antigens are selected, attention should be paid to preventing lung injury, which has been described in a case report of pulmonary injury following anti-ERBB2 CAR-T treatment (11). Several important target antigens (SDC1, MME, AFP, FOLH1, FOLR1, GPC3, ROR1, TNFRSF10B, MET, EGFR, and ERBB2) are abundant in the renal epithelialium (**Figure 1F**). In addition, MUC1, PROM1, L1CAM, and MSLN are expressed specifically in the collecting duct principal cells, whereas KIT and PSCA are expressed in the collecting duct intercalated cells. We confirmed that TNFRSF10B, MME, SDC1, IL1RAP, MUC1, PROM1, PMEL, EPHA2, MET, EPCAM, EGFR, and ERBB2 were predominantly expressed in liver epithelial cells (**Figure 1G**). We observed that TNFRSF8, CD38, and L1CAM were preferentially expressed in pancreatic Schwann cells, NCAM1 in α/β/γ/δ cells and Schwann cells, AXL in stellate and Schwann cells, FAP in α cells, PROM1 in ductal cells, and MSLN, SDC1 plus MUC1 in MUC5B+ ductal cells (**Supplementary Figure 2F**). Notably, ROR2, EGFR, GPC3, MET, EPCAM, and ERBB2 are widely expressed in various pancreatic cell populations. Furthermore, we identified the target antigen expression patterns of the trachea, bladder, esophagus, stomach, small intestine, rectum, common bile duct, and skin tissue (**Supplementary Figures 2G, H**, **3A–F**). We also established another expression profile with 64 potential target antigens from 16 tissues and organs (**Supplementary Figures 4**, **5**).

We constructed a CAR-targeted antigen expression profile of normal human tissues and organs (PBMCs, BM, SP, LN, heart, ascending aortic tissue, trachea, lung, skin tissue, kidney, bladder, esophagus, stomach, small intestine, rectum, liver, common bile duct, and pancreas) at the scRNA-seq levels. Based on these prediction analyses, we can predict, prevent, and monitor the “on-target, off-tumor toxicity” of CAR-T treatment in advance at a more detailed single-cell level.

### Inferring the CAR Safety of B Lineage-Related Target Antigens (CD19, MS4A1, CD22, TNFRSF17, SDC1, CD38, SLAMF7, and TNFRSF8)

**Figure 2:**
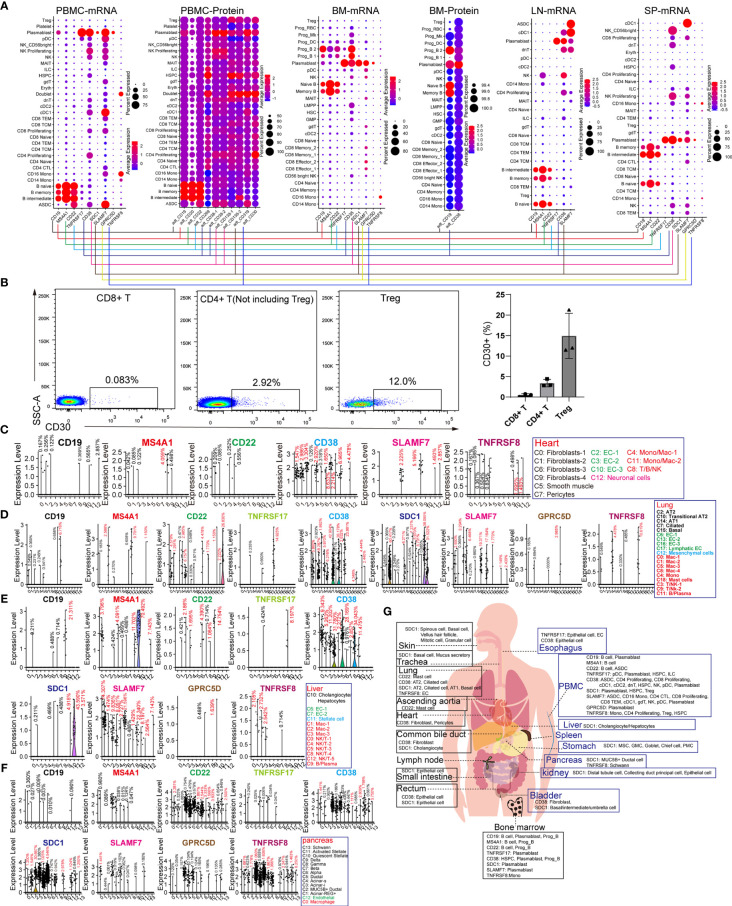
Expression patterns of B-lineage-specific antigens (CD19, MS4A1, CD22, TNFRSF17, CD38, SDC1, SLAMF7, GPRC5D, and TNFRSF8) in human normal tissues and organs. **(A)** Dot plots show the expression levels of B-lineage-specific antigens in PBMCs, BM, LN, and SP at mRNA level or protein level. **(B)** Flow cytometric analysis of abandoned human PBMCs after medical examination. Representative FACS dot plots for CD30 in normal CD8^+^ T cells (DAPI^−^CD4^−^CD8^+^), CD4^+^ T cells (DAPI^−^CD4^+^CD8^−^ but excluding Treg), and Treg cells (DAPI^−^CD4^+^CD8^−^CD127^low/−^CD25^+^). Frequency histogram of CD30+ cells in CD8^+^ T cells, CD4^+^ T cells, and Treg cells. Violin plots show the expression level of B-lineage-specific antigens in heart-derived clusters **(C)**, lung-derived clusters **(D)**, liver-derived clusters **(E)**, and pancreas-derived clusters **(F)**. **(G)** Schematic diagram of high B-lineage-specific antigen-expressing cell types in different tissues and organs.

CD19, MS4A1 (CD20), and CD22 are specific target antigens for diseases, such as relapsed/refractory large B-cell lymphoma, mantle cell lymphoma, and diffuse large B-cell lymphoma (DLBCL). CD19, SDC1 (CD138), CD38, SLAMF7, and TNFRSF17 (BCMA) were selected for targeting multiple myeloma (MM). TNFRSF8 (CD30) is a specific marker and target antigen for Hodgkin lymphoma. These lymphomas or B leukemia originate from the B-lineage. To obtain the difference “on-target, off-tumor toxicities” for adopting individualized preventive measures, we compared the expression pattern of these seven genes in normal tissues and organs. CD19 and MS4A1 had little effect on other hematopoietic lineages and were found to be ideal targets for eliminating B-lineage-derived cells than the other target antigens (**Figure 2A**). CD22 is expressed in B-lineage-derived cells and ASDC (defined by the expression of AXL and SIGLEC6), which can be identified at the mRNA and protein levels. CD38 and SLAMF7 are commonly abundant in non-B-lineages, such as pDCs, T lymphocytes, and NK cells, which is consistent with previous reports (12). Interestingly, the mRNA expression patterns of TNFRSF17, SDC1, GPRC5D, and TNFRSF8 were not identical to the corresponding protein expression levels. Then BD FACSAira II was used to analyze the expression level of TNFRSF8 in T lymphocytes derived from PBMCs (**Figure 2B**). The results showed that TNFRSF8 is highly enriched in partial Treg cells, which is inconsistent with the mRNA expression pattern. Thus, it was concluded that the protein expression pattern of CAR-target antigens is more complicated than the mRNA expression pattern displayed by scRNA-seq, and this problem can be solved by single-cell proteomics.

It is unclear whether these target antigens are also expressed in non-immune cell types. We examined the expression patterns of these genes in the heart, lung, liver, pancreas, kidney, skin, stomach, ascending aortic tissue, trachea, bladder, esophagus, small intestine, rectum, and common bile duct (**Figures 2C–F** and **Supplementary Figure 6**). Surprisingly, it was discovered that rare non-immune cells also express these B-lineage-specific genes with low mRNA levels. CD38 and TNFRSF8 are expressed in cardiac fibroblasts, SMCs, pericytes, and ECs, suggesting the potential cardiotoxicity of anti-CD38 and anti-CD30 CAR-T cells (**Figure 2C**). The CD38 and SDC1 expression patterns revealed the pulmonary toxicity and hepatotoxicity of anti-CD38 and anti-CD138 CAR-T cells (**Figures 2D, E**). Unexpectedly, these B-lineage-specific genes were also expressed in pancreatic acinar and ductal cells (**Figure 2F**). These B-lineage-specific gene expression patterns in other tissues and organs (kidney, skin, stomach, ascending aortic tissue, trachea, bladder, esophagus, small intestine, rectum, and common bile duct) revealed that the real expression patterns of CAR-target antigens were far more complex than what had been usually perceived (**Supplementary Figure 6**). The pattern diagram of cell types of these highly expressed antigens in various tissues and organs is shown in **Figure 2G**.

### Inferring the CAR Safety of AML-Related Target Antigens (CD33, CD123, and CLEC12A)

**Figure 3:**
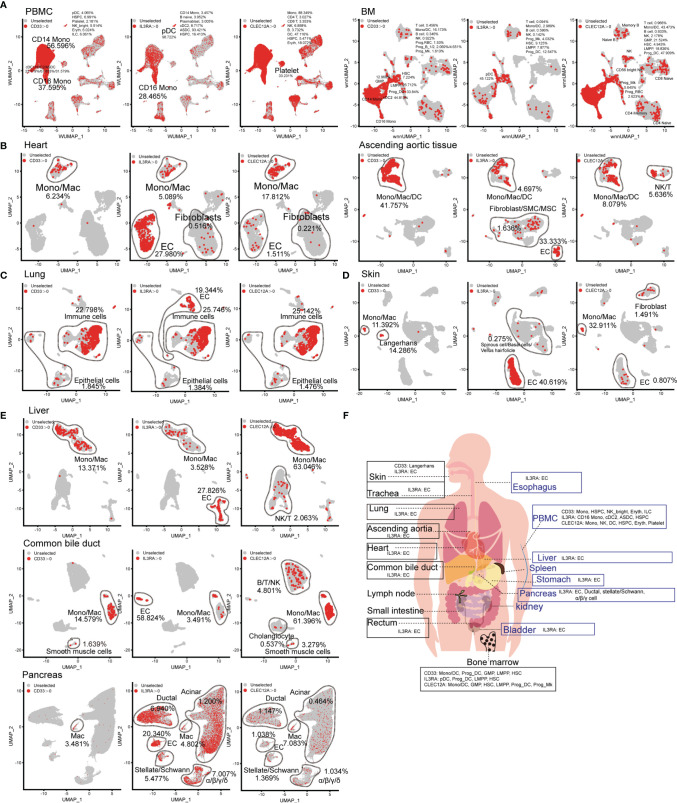
Expression pattern of AML antigens (CD33, IL3RA, and CLEC12A) in human normal tissue and organs. CD33, IL3RA, and CLEC12A-expressing proportions (expression value >0) of PBMC/BM-derived cells **(A)**, heart-derived cells, ascending aortic tissue-derived cells **(B)**, lung-derived cells **(C)**, skin-derived cells **(D)**, liver-derived cells, common bile duct-derived cells, pancreas-derived cells **(E)**, are illustrated in UMAP plots. **(F)** Schematic diagram of high AML antigen-expressing cell types in different tissues and organs.

The choice of AML-related target antigens in CAR-T immunotherapy is challenging. Therefore, we selected to analyze the expression pattern of common AML-related target antigens (CD33, CD123, and CLEC12A) in normal tissues and organs. Targeting CD33, CD123, and CLEC12A mainly damaged CD14-positive monocytes, CD16-positive monocytes, and DC populations, as well as minimal damage to other hematopoietic lineages (such as B lineages, T lymphocytes, and NK cells). It is worth noting that all these genes are expressed in partial HSPCs at the mRNA level. A more serious concern is that CLEC12A has a higher frequency than CD33 and CD123 in platelets at the mRNA level (**Figure 3A**), although a previous study showed that CLEC12A is not expressed in platelets at the protein level (13). CD123 is abundant in the ECs of various organs, such as cardiac ECs, lung ECs, skin ECs, liver ECs and urinary bladder ECs (**Figures 3B–E**, and **Supplementary Figure 7H**), and targeting this antigen may lead to endothelial-specific cross-reactivity and endothelial cell toxicity by targeting CD93 (14). A small number of cardiac fibroblasts and aortic fibroblasts/SMCs/MSCs also expressed CD123 (**Figure 3B**). CD33, CD123, and CLEC12A are present in a few lung epithelial cells (**Figure 3C**). CD33-directed CAR-T may eliminate skin Langerhans cells (**Figure 3D**), which weakens the ability of the skin to fight pathogenic microorganisms. Next, we examined the expression patterns of CD33, CD123, and CLEC12A in the urinary system, and discovered a small population of bladder fibroblasts expressing CD33 or CLEC12A (**Supplementary Figure 7H**). Compared with CD33 and CLEC12A, CD123 was predominant in multiple pancreatic cell types. We also presented the expression patterns of these genes in the SP, esophagus, trachea, stomach, small intestine, and rectum (**Supplementary Figure 7**). In conclusion, it is inferred that the probability of CD33-directed CAR-T triggering “on-target, off-tumor toxicity” is lower than that of CD123- and CLEC12A-directed CAR-T cells. Moreover, CD123-directed CAR-T might impair the systemic endothelial system, and CLEC12A-directed CAR-T is inclined to attack normal lymphocytes (**Figure 3F**).

### Inferring the CAR Safety of Solid Tumor-Related Target Antigens (GPC3, B4GALNT1, and ERBB2)

**Figure 4:**
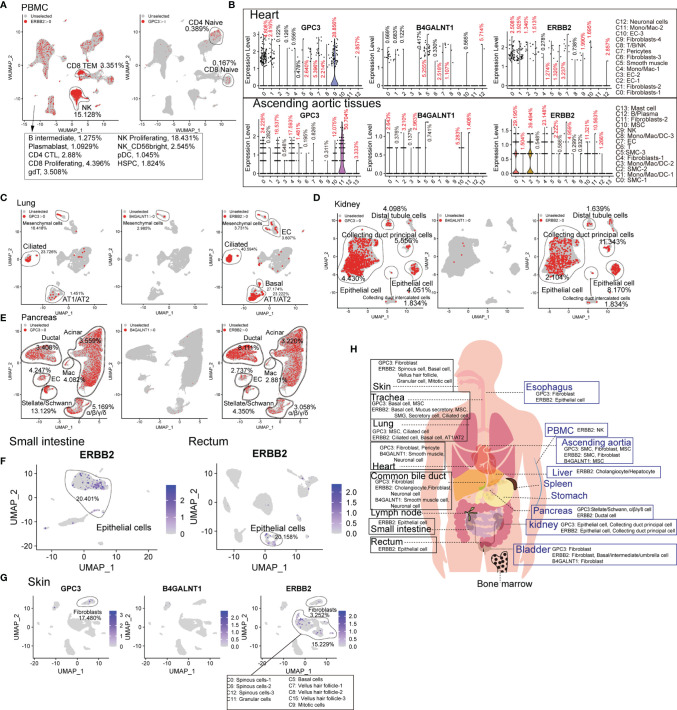
Expression patterns of solid tumor antigens (GPC3, B4GALNT1, and ERBB2) in human normal tissues and organs. **(A)** GPC3 and ERBB2-expressing proportions (expression value >0) of PBMC-derived cells. **(B)** Violin plots show the expression levels of GPC3, B4GALNT1, and ERBB2 in heart-derived clusters and ascending aortic tissue-derived cells. GPC3, B4GALNT1, and ERBB2-expressing proportions (expression value >0) of lung-derived cells **(C)**, kidney-derived cells **(D)**, and pancreas-derived cells **(E)**. **(F)** UMAP plots show the expression level of ERBB2 in small intestine-derived cells and rectum-derived cells. **(G)** UMAP plots show the expression levels of GPC3, B4GALNT1, and ERBB2, in skin-derived clusters. **(H)** Schematic diagram of high GPC3/B4GALNT1/ERBB2-expressing cell types in different tissues and organs.

Compared to hematologic malignancies, the understanding of the “on-target, off-tumor toxicity” of CAR therapies for solid tumors has been lagging. The common target antigens of solid tumor types exist on normal cells, leading to severe “on-target, off-tumor toxicity”, which constrains their application. Therefore, we analyzed the common target antigens (GPC3, B4GALNT1, and ERBB2 [HER2]) in various tissues and organs at the scRNA-seq level. ERBB2 and GPC3 are expressed in immune cell types. ERBB2-directed CAR-T cells may presumably eliminate ERBB2-positive CD8^+^ TEM, NK cells, and bone marrow cells (**Supplementary Figure 8**). Moreover, GPC3-directed CAR-T cells may attack a portion of naïve T lymphocytes (**Figure 4A**). GPC3 is abundant in cardiac and aortic fibroblasts, B4GALNT1 in cardiac and aortic SMCs, and ERBB2 in cardiac fibroblasts and aortic SMCs (**Figure 4B**). B4GALNT1 was hardly observed in lungs, kidneys, and pancreas, while GPC3 and ERBB2 are commonly expressed in normal cells, such as AT1, AT2, basal cells, kidney epithelial cells, and various pancreatic cells (**Figures 4C–E**). ERBB2 also exists in normal epithelial cells of the small intestine and rectum, at high levels, and in liver hepatocytes/cholangiocytes (**Figure 4F** and **Supplementary Figure 8B**). Predictably, targeting these antigens may impair the function of skin because skin fibroblasts express GPC3, skin pericytes express B4GALNT1, and hair follicle-related cells express ERBB2 at high levels (**Figure 4G**). In addition, we acquired the expression pattern of these genes in the common bile duct, bladder, trachea, and esophagus, from which it was identified that these antigens extensively exist in normal tissues and organs (**Supplementary Figure 8C**). Eventually, it is known that B4GALNT1 is a relatively ideal target, but targeting GPC3 and ERBB2 might lead to severe “on-target, off-tumor toxicity” in some tissues (**Figure 4H**).

### The Expression Levels of Targets in Normal Cells and Malignant Cells

**Figure 5:**
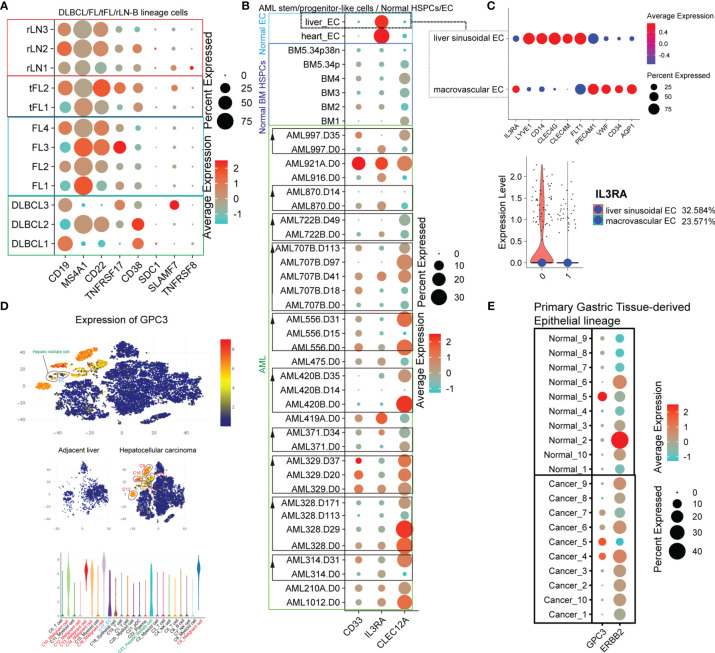
The difference of expression patterns of solid tumor antigens (GPC3, B4GALNT1, and ERBB2) in malignant cells and nonmalignant cells. **(A)** Dot plot shows the expression level of target antigens (CD19, MS4A1, CD22, TNFRSF17, CD38, SDC1, SLAMF7, and TNFRSF8) in B lineage-related cells derived from lymphomas (DLBCL, FL, and tFL) and rLN. **(B)** Dot plot shows the expression level of target antigens (CD33, IL3RA, and CLEC12A) in hematopoietic stem/progenitor-like cells obtained from BM samples of AML patients and healthy donors, and in normal liver/heart EC clusters. “AML314.D31” represents the sample of patient AML314 after 31 days of treatment. BM5.34p means the sample of BM CD34-positive cells derived from healthy donor BM5, and BM5.34p38n means the sample of BM CD34-positive and CD38-negative cells derived from healthy donor BM5. **(C)** The expression level of IL3RA in liver sinusoidal endothelial cells and macrovascular endothelial cells. **(D)** GPC3 expression levels in adjacent liver and hepatocellular carcinoma at scRNA-seq level. **(E)** GPC3 and ERBB2 expression levels of the epithelial lineages in normal primary gastric tissues and gastric cancers.

Many CAR-T therapies, in fact, efficiently killed tumor cells expressing high levels of target antigens but not tumor cells or normal cells with lower levels of target antigens (15–21). The CAR-T anti-tumor activity and “on-target, off-tumor toxicity” are dependent on the target antigen density. Locoregional HER2-specific CAR-T injection through intra-CNS delivery was well tolerated and showed no evidence of “on-target, off-tumor toxicity” (22). Another study also showed that side effects could be avoided by optimizing CAR design, decreasing the dose of CAR-T cells, and improving the treatment plan (such as omitting post-infusion IL-2 and lymphodepleting chemotherapy) (23). We compared several tumor types with normal tissues at scRNA-seq levels to provide a broader and more realistic perspective of “on-target, off-tumor toxicity”. First, we compared the target antigen expression levels of reactive non-malignant lymph nodes (rLN) and malignant lymph nodes (DLBCL, follicular lymphomas [FL], and transformed FL [tFL])-derived B-lineage cells (**Supplementary Figure 9A**). The target antigen expression levels of non-malignant and malignant cells were highly variable (**Figure 5A**). We found that CD19 expression levels were significantly decreased in some samples (DLBCL2, 1/3 samples; FL1 and FL3, 2/4 samples; tFL1, 1/2 samples), MS4A1 in DLBCL1 and DLBCL3 (2/3 samples), CD22 in some DLBCL samples (DLBCL1 and DLBCL3, 2/3 samples) and FL1 (1/4 samples). In addition, we also found that TNFRSF17 was significantly increased in DLBCL1, FL3, and tFL2 compared with the rLN samples, CD38 in some DLBCL samples (DLBCL1 and DLBCL2, 2/3), and SLAMF7 in DLBCL3. Next, we compared the AML-related antigen (CD33, IL3RA, and CLEC12A) expression levels of normal EC populations from the heart and liver, normal BM-derived HSPC, and AML patient-derived progenitor-like cells (**Supplementary Figure 9B**). CLEC12A was strongly positive in almost all AML samples (**Figure 5B**). We also observed significant upregulation of CD33 and IL3RA in almost all AML samples compared to the normal BM HSPC. Interestingly, the target antigen (CD33, IL3RA, and CLEC12A) expression levels in AML progenitor-like populations in AML patients during treatment were highly variable. Strikingly, the IL3RA expression level in the heart and lung-derived EC populations was significantly higher than that of hematopoietic cells at the mRNA level, which might lead to vascular leak when targeting IL3RA (24). Moreover, the expression levels of IL3RA in different subsets, such as liver sinusoidal EC and macrovascular EC, are different, which remind us that there exist many uncertainties of “on-target, off-tumor toxicity” because of the cell heterogeneity and thus need to further identification (**Figure 5C**). To further confirm the expression level of the target antigen in solid tumors, GPC3 was examined in hepatocellular carcinoma (HCC) and adjacent liver (25). GPC3 is highly expressed in malignant cell subsets of HCC (**Figure 5D**). Disturbingly, some normal hepatic stellate cells and EC also express GPC3 at a high level. Moreover, we examined GPC3 and ERBB2 expression levels in gastric cancer (GC) (**Supplementary Figure 9C** and **Figure 5E**). These primary gastric tissue-derived epithelial cells express GPC3 at high levels in some GC samples (Cancer_4, Cancer_5, Cancer_6, and Cancer_7, 4/10) and a normal tissue sample (Normal_5, 1/10). ERBB2 is widely expressed in epithelial populations of both normal samples (Normal_2, Normal_3, Normal_5, Normal_6, Normal_10, 5/10) and gastric cancer samples (Cancer_1, Cancer_2, Cancer_3, Cancer_4, Cancer_6, Cancer_7, Cancer_8, Cancer_9, and Cancer_10, 9/10) at high levels. Taken together, although the proportion and expression level of some target antigens in tumors usually increases, there are still some normal cells that express these antigens at high levels. This expression pattern demonstrates that identifying the difference in antigen expression levels in the same patient before CAR-T treatment is critically important and may help reduce the probability of “on-target, off-tumor toxicity.”

## Conclusion

With the rapid increase in CAR-T clinical trials, numerous treatment-related side effects have been observed, which severely restricts the further application of CAR-T cells. One of the most important CAR-T treatment-related side effects is “on-target, off-tumor toxicity”, Substantial evidence of “on-target, off-tumor toxicity” has been shown in CAIX-directed CAR-T (6), FAP-directed CAR-T (7), CD19-directed CAR-T (2), CD133-directed CAR-T (26), HER2-directed CAR-T (27), EGFR-directed CAR-T (28), CD38-directed CAR-T (3), CD138-directed CAR-T (29), CD33-directed CAR-T (30–32), and CD123-directed CAR-T (33, 34). This reminds us that most of the existing CAR target antigens are not as highly specific as we previously expected.

To determine which normal cells may be improperly targeted by CAR-T cell therapies, we chose 18 normal tissues/organs and analyzed CAR target antigen expression patterns *via* publicly available scRNA-seq datasets. scRNA-seq is a powerful tool for the understanding of the different cell subsets (35), analyzing rare cell types (36), and exploring the complex regulatory networks and developmental trajectories (37, 38). Finally, we obtained the expression patterns of 121 target antigens in normal tissues or organs at the single-cell level. Based on this, predictions can be made about the “on-target, off-tumor toxicity” of CAR-T therapies, which can guide the minimization or monitoring of these side effects. Based on the expression patterns of target antigens that we have identified, clinical researchers can comprehend the antigens expressed in normal cell types, especially in special cell types of tissues and organs, such as CD22-expressing mast cells in ascending aortic tissue and EPCAM-expressing erythroid progenitors. B-lineage-related target antigens (CD19, MS4A1, CD22, TNFRSF17, CD38, SLAMF7, and TNFRSF8) not only exist in B lineage-derived normal or malignant tumor cells but are also expressed in other immune cell types (CD22/CD38/SLAMF7-expressing ASDC, CD38-expressing proliferating T lymphocytes, and SLAMF7-expressing cDC1) and non-immune cell types (CD38-expressing cardiac fibroblasts, SDC1-expressing AT1/AT2/basal cells, and CD22/CD38/SDC1/TNFRSF8-expressing pancreatic acinar/ductal cells) at a certain frequency. CD123 is enriched in the EC population and pancreatic cells, suggesting that CD123-directed CAR therapy may cause vascular endothelial dysfunction and pancreatic injury. Compared with CD123 and CLEC12A, targeting CD33 may have reduced damage to non-immune cell types. GPC3 and ERBB2, as common solid tumor target antigens, are frequently diffused in various tissues and organs; therefore, reducing the damage to important organs while targeting these antigens is a top priority. Other target antigens have also shown potential in CAR-T therapy, such as IGF1R (39, 40), CD1A (41, 42), CCR9, and CXCR4. Our results showed that IGF1R is widely expressed in various lineages from three germ layers, while the expression of CD1A, CCR9, and CXCR4 is relatively limited to immune cell types (**Supplementary Figure 10**). Moreover, we should solve the problems of self-activation and fratricide of CAR-T cells before targeting these antigens (CCR9 and CXCR4). It should be noted that, although scRNA-seq can provide reliable information, the expression pattern of antigens at the protein level is complicated. These could be solved using single-cell proteomics technology.

Compared to their normal counterparts, some antigens of malignant cells from different patients are highly variable because of drug-specific selection and genetic variation (43, 44). For example, CD38 was consistently downregulated in the emerging resistant clones in response to a combined therapy regimen (daratumumab, carfilzomib, lenalidomide and dexamethasone) (45). Our results also defined the variable expression of target antigens in normal and malignant cells, which suggests that identifying the difference in target antigen expression levels between malignant cells and their normal counterparts before CAR-T treatment is important to avoid unnecessary “on-target, off-tumor toxicity”. Indeed, most side effects observed during CAR-T treatment, such as cytokine release syndrome and immune effector cell-associated neurotoxicity syndrome, can be reversed if they are treated promptly. The “on-target, off-tumor toxicity” of CD19-directed CAR-T cells, such as B-cell dysplasia and hypogammaglobulinemia, can be reversed after a few months of CAR-T cell infusion because of the existence of HSPC and plasma cells. This evidence indicates that “on-target, off-tumor toxicity” of CAR-T cell therapy can be reduced by accurate treatment, and the physiological homeostasis can be restored because of the existence of stem/progenitor cells and antigen low-expressing cells.

In conclusion, we developed a comprehensive single-cell atlas for target antigens of CAR therapy in normal tissues and organs, which helped us capture antigen-expressing rare cell types missed in the assessment of bulk tissues.

## Materials and Methods

### Flow Cytometry Analysis

Venous blood samples from healthy donors were collected in EDTA anticoagulant tubes and stored at 4°C. PBMCs were isolated by density gradient centrifugation using Ficoll–Paque PLUS (Cat No. 17-1440-03, GE Healthcare). PBMCs were blocked by FcR (CD16/32) Blocking Reagent (Cat No. 130-059-901, Miltenyi Biotec) antibody, and stained with the following antibodies: APC/Cyanine7 anti-human CD4 antibody (A161A1, Cat No. 357415, Biolegend), FITC anti-human CD8a antibody (RPA-T8, Cat No. 301050, Biolegend), PE-Cyanine 7 anti-human CD127 antibody (A7R34, Cat No. 25-1271-82, eBioscience), PerCP/Cyanine5.5 anti-human CD25 antibody (BC96, Cat No. 302626, Biolegend), and APC anti-human CD30 antibody (BY88, Cat No. 333910, Biolegend). Finally, the cells were resuspended in 400 ul 0.1 ug/ml DAPI solution (Cat No. C0060, Salarbio) and analyzed using an Arial II cytometer (BD Biosciences). Flow cytometry data were analyzed using FlowJo (Three Star, Ashland OR). Percentage data were presented as mean ±  SD using GraphPad Prism.

### scRNA-seq Datasets

The scRNA-seq datasets of the trachea (GSM4850591), stomach (GSM4850590), spleen (GSM4850589), small intestine (GSM4850588), skin (GSM4850587), rectum (GSM4850586), lymph node (GSM4850583), liver (GSM4850582), heart (GSM4850581), esophagus (GSM4850580), common bile duct (GSM4850579), and bladder (GSM4850577), were acquired from the GEO database (Accession NO. GSE159929) (46). The scRNA-seq datasets of the ascending aortic tissue (GSM4704931, GSM4704932, and GSM4704933) were obtained from GSE155468. The scRNA-seq datasets of the normal lung samples were extracted from GSE135893 by performing “subset(x = lung, subset = orig.ident == c(“F00409”,”F01157”,”F01174”, “F01365”, ”F01366”, ”F01367”, ”F01394”, ”HD65”, ”HD66”, ”HD67”, ”HD68”, ”HD70”))” (47). The multimodal PBMC reference dataset was downloaded from https://atlas.fredhutch.org/data/nygc/multimodal/pbmc_multimodal.h5seurat. The multimodal human bone marrow mononuclear (BMNC) reference dataset was obtained by performing “InstallData (“bmcite”)”, and “LoadData(ds = “bmcite”)” in R. The human pancreas dataset was downloaded from http://singlecell.charite.de/pancreas/Adult_Pancreas/adult_pancreas_2020.rds (48).

### Quality Control

Cells from the LN, SP, stomach, small intestine, rectum, esophagus, common bile duct, skin, trachea, bladder, heart, and liver were filtered with a gene expression number per cell between 200 and 10,000, and the mitochondrial percentage per cell was below 15. Cells from the ascending aortic tissue, kidney, and lung were filtered with a gene expression number per cell between 200 and 10,000, with a mitochondrial percentage below 10, 50, and 20, respectively.

### Data Processing

We mapped the LN and SP scRNA-seq datasets to Satijalab’s reference of 162,000 PBMCs measured with 228 antibodies in Seurat V4 (49). Other datasets also were processed in Seurat V4, and the “NormalizeData” function was used to normalize the expression matrix. Then “FindVariableFeatures”, “ScaleData”, and “RunPCA” were used to process the datasets. Clusters were calculated using the FindClusters function with a resolution of 0.5 and visualized using the uniform manifold approximation and projection (UMAP) dimensional reduction method. All major cell clusters were identified by feature genes, as shown in **Supplementary Figure 1B**. “DotPlot” function, “VlnPlot” function, “FeaturePlot” function, and “DimPlot” function with “cells.highlight=WhichCells(object, expression =gene> 0)” were used to visualize gene expression.

### Comparison of Gene Expression Profiles of AML Malignant Progenitors With Normal BM HSPCs and Liver/Heart EC

Single-cell gene expression data for AML BM cells and normal BM cells were obtained from GSE116256 (50). Sixteen AML patient-derived 34 samples, four healthy donor-derived five samples, healthy donor-derived liver EC (extracted from cluster 6/7 in **Figure 1G**), and healthy donor-derived heart EC (extracted from cluster 2/3/10 in **Figure 1D**) were combined by the function “merge” in Seurat V4. Cells from each dataset were filtered with a gene expression number per cell between 200 and 10,000, with a mitochondrial percentage below 10. The batch effect was removed using the “SCTransform(object, vars.to.regress = “percent.mt”)” and “RunHarmony(object, group.by.vars= “orig.ident”)” function. Clusters were calculated using the FindClusters function with a resolution of 0.5 and visualized using the UMAP dimensional reduction method. Then, the stem/progenitor-like subpopulations and EC subpopulations were extracted by “subset(object, idents = c(0,3,10,11,13,16,18,21,8,19))”. Finally, the expression level was compared using the “Dotplot” function with “group.by = “orig.ident”. Liver EC population was calculated using FindClusters function with a resolution of 0.5, and the representative genes (liver sinusoidal EC: LYVE1, CD14, CLEC4G, and CLEC4M; macrovascular EC: PECAM1, VWF, CD34, and AQP1) were presented (51).

### Comparison of Gene Expression Profiles of Lymphomas With rLN

Single-cell gene expression data of lymphomas (three DLBCL samples, four FL samples, and two tFL samples) and rLN (three samples) were obtained from https://www.zmbh.uni-heidelberg.de/Anders/scLN-index.html (52). These datasets were merged in Seurat V4 and filtered with a mitochondrial percentage below 10. “SCTransform” and “RunHarmony” functions were used to remove the batch effect as mentioned above. Clusters were calculated using the FindClusters function with a resolution of 0.5, and the B lineage-related subpopulations were extracted by “subset(object, idents = c(0,1,3,4,5,8,10,11,12,13,14,15,17,18,19,20,21,22))”. Finally, the expression level was compared using the “Dotplot” function with “group.by = “orig.ident”.

### Comparison of Gene Expression Profiles of Hepatocellular Carcinoma With Adjacent Liver

We compared the GPC3 expression level of hepatocellular carcinoma with the adjacent liver on an online website (https://db.cngb.org/PRHCCdb), which is shared by the laboratory of Jia Fan (25).

### Comparison of Gene Expression Profiles of Gastric Cancer With Healthy Primary Gastric Tissue

The datasets of ten gastric cancer samples (GSM5573467, GSM5573468, GSM5573470, GSM5573472, GSM5573473, GSM5573475, GSM5573477, GSM5573478, GSM5573479, and GSM5573481) and ten healthy primary gastric tissues (GSM5573466, GSM5573469, GSM5573471, GSM5573474, GSM5573476, GSM5573486, GSM5573488, GSM5573490, GSM5573490, and GSM5573496) were downloaded from GSE183904, and combined by the function “merge” in Seurat V4. Cells from each dataset were filtered with a gene expression number per cell between 200 and 10000, with a mitochondrial percentage below 20. The batch effect was removed using the “SCTransform(object, vars.to.regress = “percent.mt”)” and “RunHarmony(object, group.by.vars = “orig.ident”)” functions. Clusters were calculated using the FindClusters function with a resolution of 0.5 and visualized using the UMAP dimensional reduction method. Then the epithelial subpopulations were extracted by “subset(object, idents = c(1,4,8,10,16,23,24))”. Finally, the expression level was compared using “Dotplot” function with “group.by = “orig.ident”.

## Data Availability Statement

The datasets presented in this study can be found in online repositories. The names of the repository/repositories and accession number(s) can be found in the article/**Supplementary Material**.

## Ethics Statement

The studies involving human participants were reviewed and approved by the Research and Clinical Trial Ethics Committee of the First Affiliated Hospital of Zhengzhou University. The patients/participants provided their written informed consent to participate in this study.

## Author Contributions

YZ and RQG designed and performed the experiments, analyzed the data, and wrote the paper. YML, WC, FW, XX, YDL, and XW performed experiments and analyzed data. RQG, RG, and ZJ initiated the study and organized, designed, and wrote the paper. All authors contributed to the article and approved the submitted version.

## Funding

This work was supported by the National Natural Science Foundation of China (No. 82100240, RQG; No. U1804192, YML), the China Postdoctoral Science Foundation (2021M692929, RQG), the Key scientific research projects of colleges and universities in Henan Province (No. 225320016, RQG; No. 19A320046, YML), the Postdoctoral Research Start-up Funding of Henan Province (202001006, RQG), the Joint Co-construction Project of Henan Medical Science and Technology Research Plan (LHGJ20200280, RQG), the Postdoctoral Research Start-up Funding of the First Affiliated Hospital of Zhengzhou University (RQG), the Medical Science and Technology Research Project Of Henan Province (No. 2018020118, YZ), and the Key Research and Development and Promotion Project of Henan province (RQG).

## Conflict of Interest

The authors declare that the research was conducted in the absence of any commercial or financial relationships that could be construed as a potential conflict of interest.

## Publisher’s Note

All claims expressed in this article are solely those of the authors and do not necessarily represent those of their affiliated organizations, or those of the publisher, the editors and the reviewers. Any product that may be evaluated in this article, or claim that may be made by its manufacturer, is not guaranteed or endorsed by the publisher.

## Supplementary Material

Supplementary Figure 1 Identification of feature genes of different clusters in normal tissues and organs. **(A)** UMAP projections of PBMC/BM-derived cells. **(B)** Expression levels of representative genes for different cell types from 13 tissues and organs (heart, ascending aortic tissue, trachea, lung, skin, esophagus, stomach, small intestine, rectum, kidney, bladder, liver, and common bile duct).

Supplementary Figure 2 Identification of the expression pattern of CAR target antigens in normal tissues and organs (PBMCs, BM, LN, SP, trachea, and bladder). Dot plot shows the protein expression levels of CAR target antigens in PBMCs **(A)** and BM **(B)**. UMAP projections of LN-derived cells **(C)**, SP-derived cells **(D)**, pancreas-derived cells **(E)**, ascending aortic tissue-derived cells **(F),** trachea-derived cells **(G)**, and bladder-derived cells **(H)**, colored by clusters, and dot plots showing the expression levels of CAR target antigens in different clusters.

Supplementary Figure 3 UMAP projection of esophagus-derived cells **(A)**, stomach-derived cells **(B)**, small intestine-derived cells **(C)**, rectum-derived cells **(D)**, common bile duct-derived cells **(E)**, and skin-derived cells **(F)**, and dot plots showing the expression levels of CAR target antigens in different clusters.

Supplementary Figure 4 The expression patterns of 64 potential target antigens. **(A)** The gene list of 64 potential target antigens. Dot plot shows the expression levels of 64 potential target antigens in PBMCs **(B)**, SP **(C)**, BM **(D)**, LN **(E)**, heart **(F)**, ascending aortic tissue **(G)**, trachea **(H)**, lung **(I)**, and skin **(J)**.

Supplementary Figure 5 The expression patterns of 64 potential target antigens in esophagus **(A)**, stomach **(B)**, small intestine **(C)**, rectum **(D)**, liver **(E)**, common bile duct **(F)**, and pancreas **(G)**.

Supplementary Figure 6 Violin plots show the expression levels of B-lineage-specific antigens in kidney-derived clusters **(A)**, skin-derived clusters **(B)**, stomach-derived clusters **(C)**, ascending aortic tissue-derived clusters **(D)**, trachea-derived clusters **(E),** bladder-derived clusters **(F)**, esophagus-derived clusters **(G)**, small intestine-derived clusters **(H)**, rectum-derived clusters **(I)**, and common bile duct-derived clusters **(J)**.

Supplementary Figure 7 Expression patterns of AML antigens (CD33, IL3RA, and CLEC12A) in human normal tissues and organs. **(A)** UMAP plots show the protein expression level of IL3RA in PBMC/BM-derived clusters. CD33, IL3RA, and CLEC12A-expressing proportions (expression value > 0) of SP-derived cells **(B)**, esophagus-derived cells **(C)**, trachea-derived cells **(D)**, stomach-derived cells **(E)**, small intestine-derived cells **(F)**, rectum-derived cells **(G),** kidney-derived cells, bladder-derived cells **(H)**, are illustrated in UMAP plots.

Supplementary Figure 8 Expression levels of solid tumor antigens (GPC3, B4GALNT1, and ERBB2) in human normal tissues and organs. **(A)** GPC3 and ERBB2-expressing proportions (expression value > 0) of BM-derived cells. **(B)** Violin plot shows the expression level of ERBB2 in liver-derived clusters. **(C)** Violin plots indicate the expression levels of GPC3, B4GALNT1, and ERBB2 in common bile duct-derived clusters, bladder-derived clusters, trachea-derived clusters, and esophagus-derived clusters.

Supplementary Figure 9
**(A)** UMAP projection of lymphoma- and rLN-derived cells, and dot plot showing the expression levels of representative genes for different cell types. **(B)** UMAP projection of AML- and healthy donor-derived BM cells, and dot plot showing the expression levels of representative genes for different cell types. **(C)** UMAP projection of single cells of normal primary gastric tissue and gastric cancer, and dot plot showing the expression levels of representative genes for different cell types.

Supplementary Figure 10
**(A)** Dot plots show the expression levels of target antigens (IGF1R, CD1A, CCR9, and CXCR4) in PBMCs, BM, Trachea, lung, stomach, esophagus, kidney, small intestine, rectum, liver, bladder, common bile duct, pancreas, heart, ascending aortic tissue, and skin. **(B)** Summarization of high IGF1R/CD1A/CCR9/CXCR4-expressing subpopulations in various tissues and organs.
