# Supplementary figures and images for: Single-Cell Analysis of Target Antigens of CAR-T Reveals a Potential Landscape of “On-Target, Off-Tumor Toxicity”
Source: Front Immunol. 2021 Dec 16;12:799206. doi: 10.3389/fimmu.2021.799206 (PMC8716389; doi:10.3389/fimmu.2021.799206)

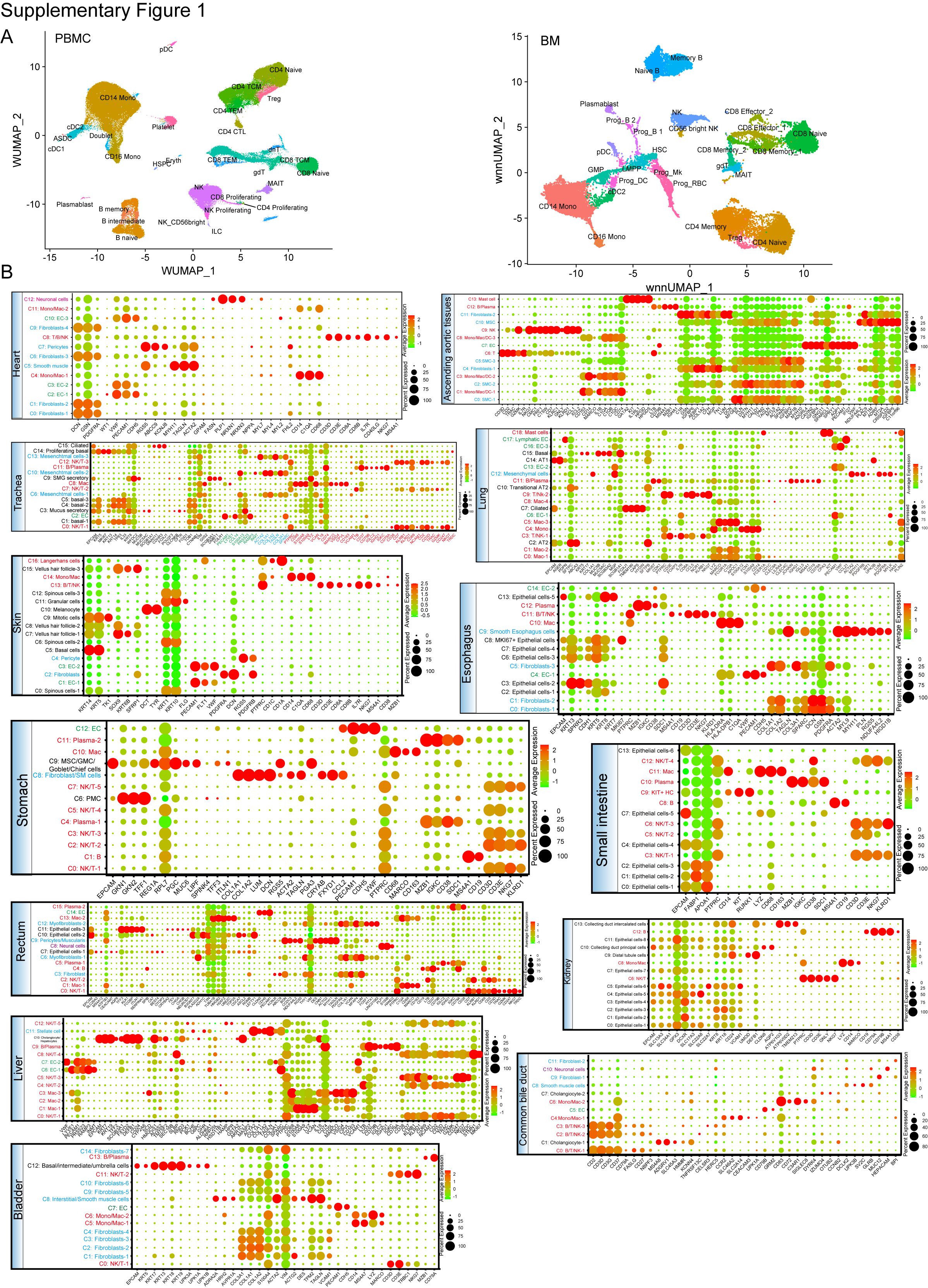

Supplement: Supplementary Figure 1 — Identification of feature genes of different clusters in normal tissues and organs. (A) UMAP projections of PBMC/BM-derived cells. (B) Expression levels of representative genes for different cell types from 13 tissues and organs (heart, ascending aortic tissue, trachea, lung, skin, esophagus, stomach, small intestine, rectum, kidney, bladder, liver, and common bile duct). [file Image_1.tif]

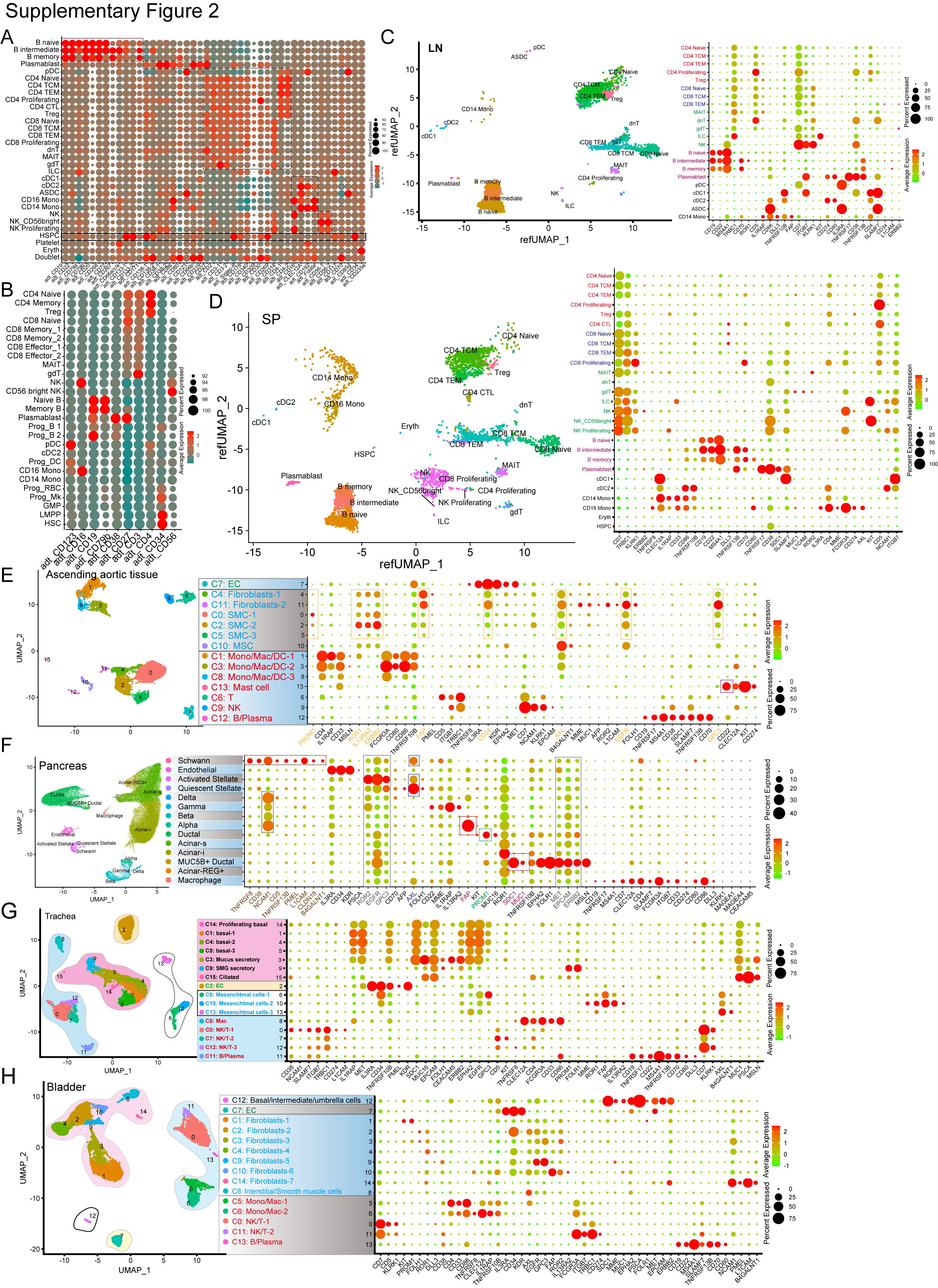

Supplement: Supplementary Figure 2 — Identification of the expression pattern of CAR target antigens in normal tissues and organs (PBMCs, BM, LN, SP, trachea, and bladder). Dot plot shows the protein expression levels of CAR target antigens in PBMCs (A) and BM (B). UMAP projections of LN-derived cells (C), SP-derived cells (D), pancreas-derived cells (E), ascending aortic tissue-derived cells (F), trachea-derived cells (G), and bladder-derived cells (H), colored by clusters, and dot plots showing the expression levels of CAR target antigens in different clusters. [file Image_2.tif]

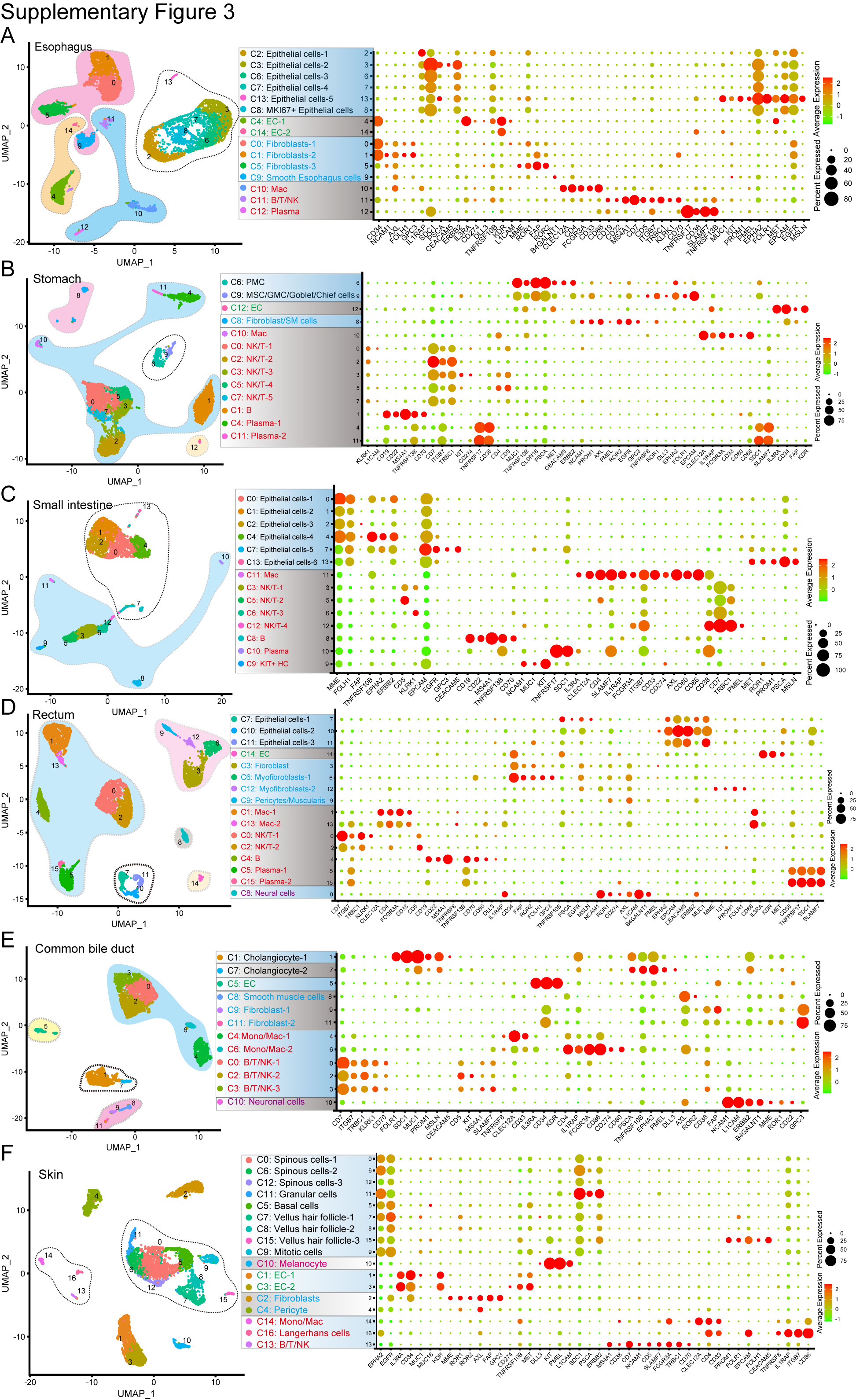

Supplement: Supplementary Figure 3 — UMAP projection of esophagus-derived cells (A), stomach-derived cells (B), small intestine-derived cells (C), rectum-derived cells (D), common bile duct-derived cells (E), and skin-derived cells (F), and dot plots showing the expression levels of CAR target antigens in different clusters. [file Image_3.tif]

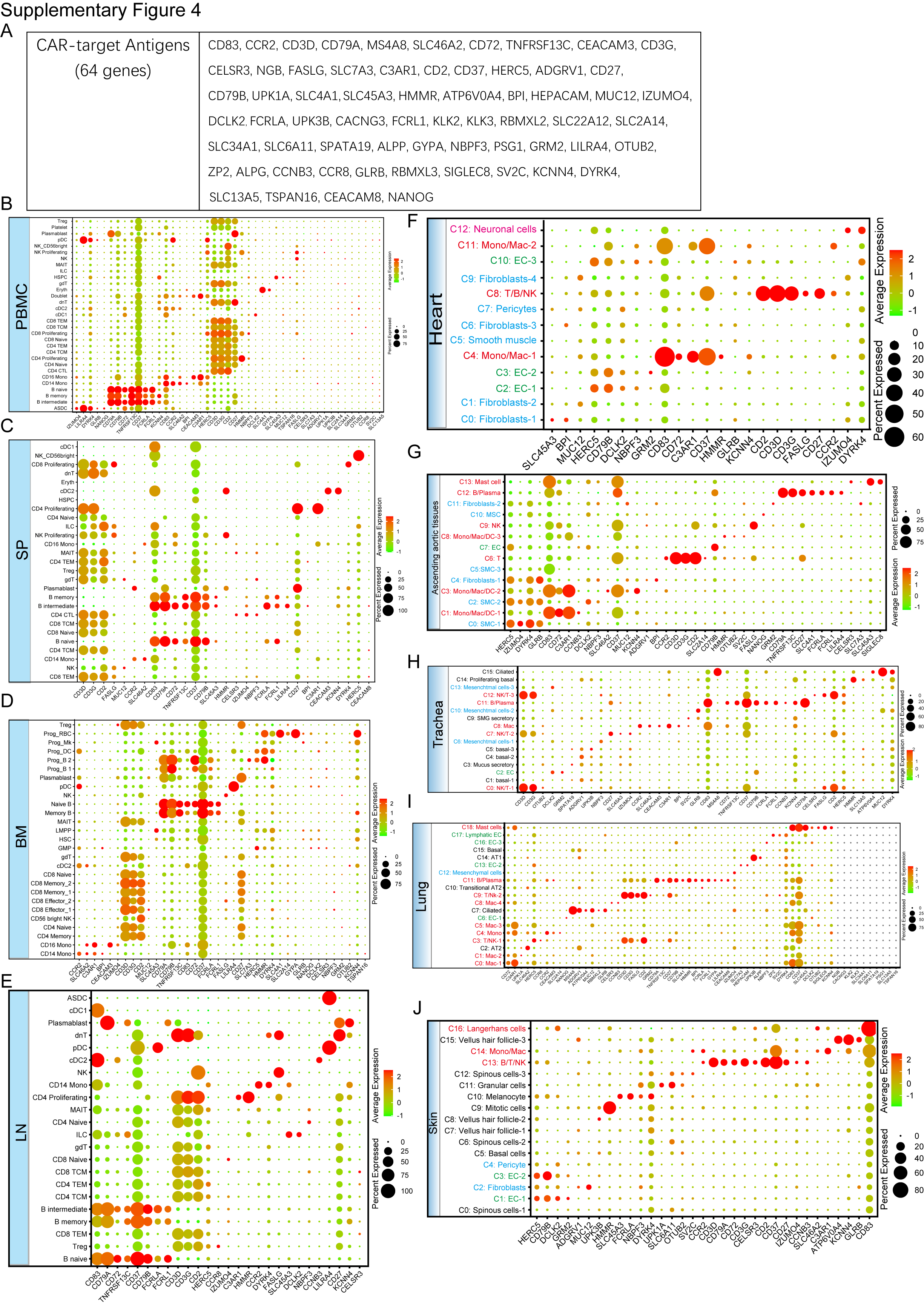

Supplement: Supplementary Figure 4 — The expression patterns of 64 potential target antigens. (A) The gene list of 64 potential target antigens. Dot plot shows the expression levels of 64 potential target antigens in PBMCs (B), SP (C), BM (D), LN (E), heart (F), ascending aortic tissue (G), trachea (H), lung (I), and skin (J). [file Image_4.tif]

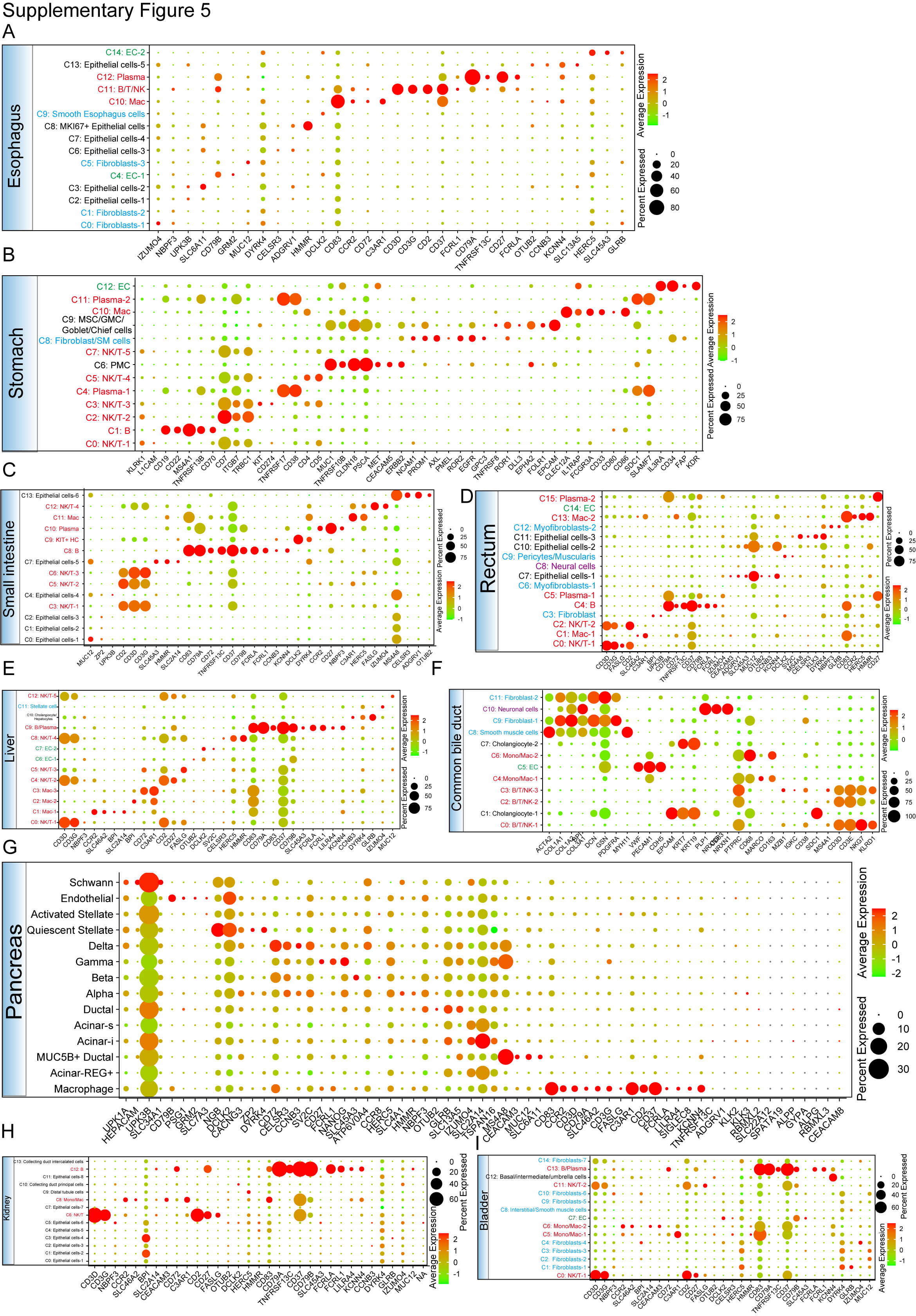

Supplement: Supplementary Figure 5 — The expression patterns of 64 potential target antigens in esophagus (A), stomach (B), small intestine (C), rectum (D), liver (E), common bile duct (F), and pancreas (G). [file Image_5.tif]

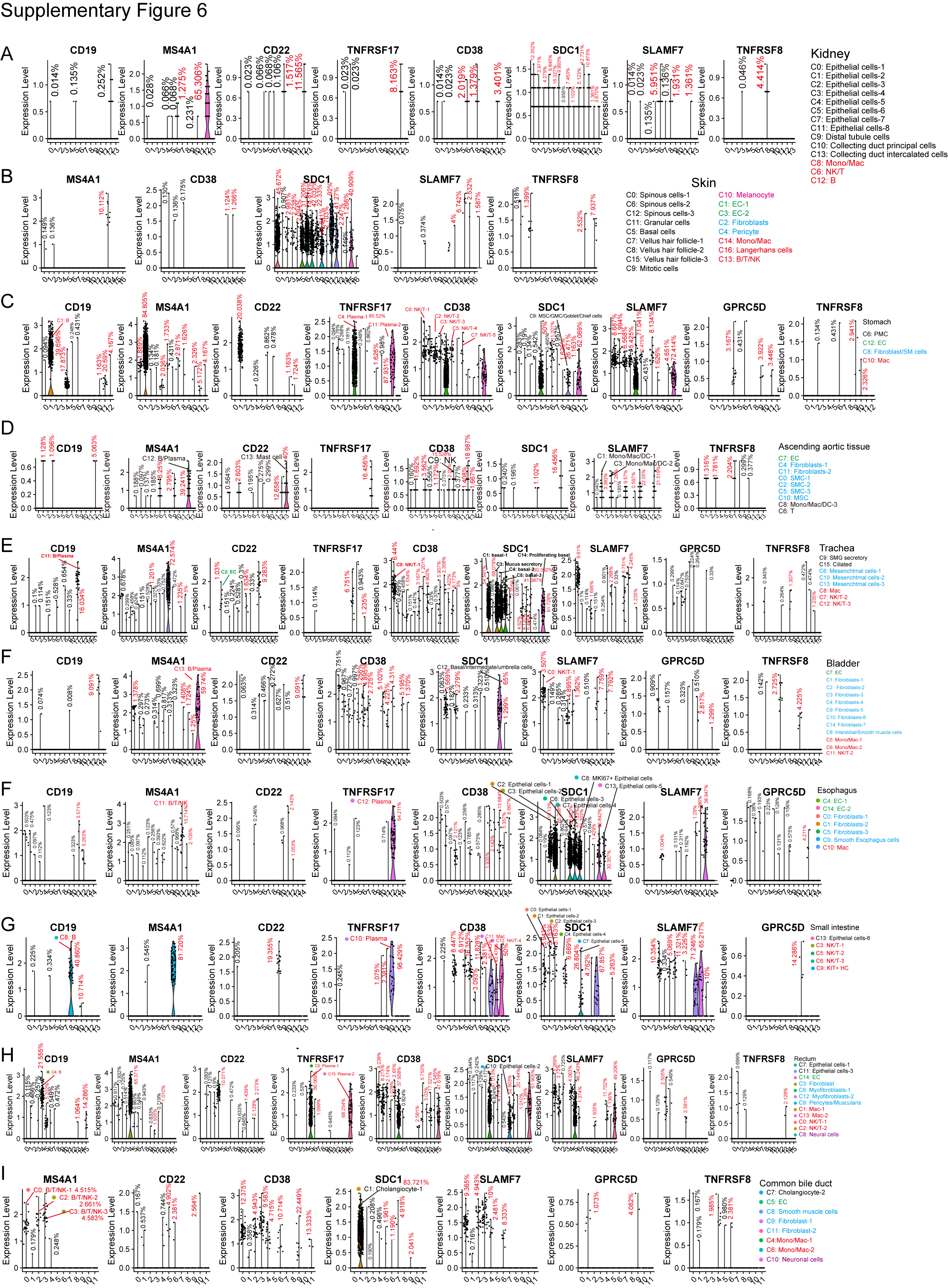

Supplement: Supplementary Figure 6 — Violin plots show the expression levels of B-lineage-specific antigens in kidney-derived clusters (A), skin-derived clusters (B), stomach-derived clusters (C), ascending aortic tissue-derived clusters (D), trachea-derived clusters (E), bladder-derived clusters (F), esophagus-derived clusters (G), small intestine-derived clusters (H), rectum-derived clusters (I), and common bile duct-derived clusters (J). [file Image_6.tif]

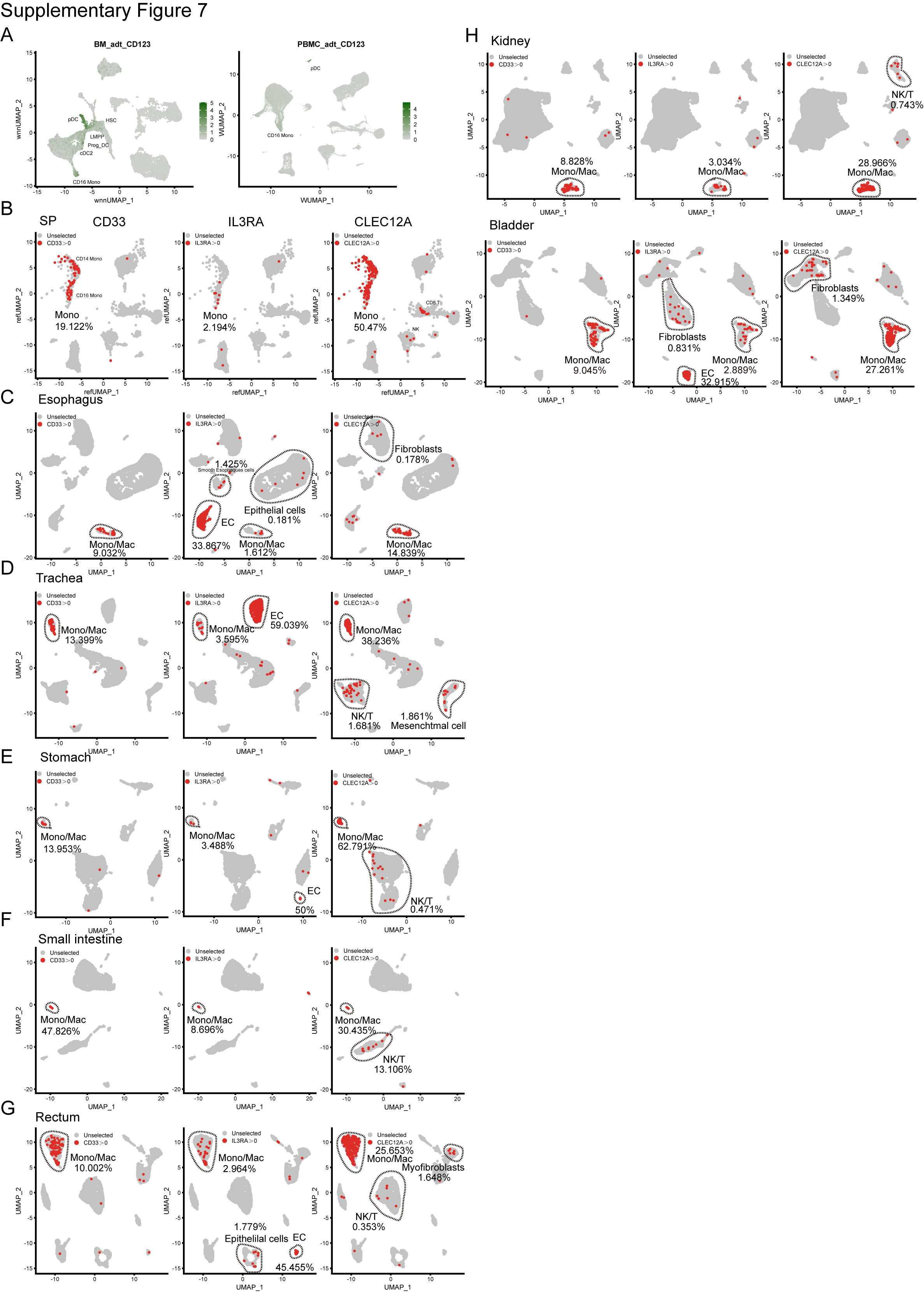

Supplement: Supplementary Figure 7 — Expression patterns of AML antigens (CD33, IL3RA, and CLEC12A) in human normal tissues and organs. (A) UMAP plots show the protein expression level of IL3RA in PBMC/BM-derived clusters. CD33, IL3RA, and CLEC12A-expressing proportions (expression value > 0) of SP-derived cells (B), esophagus-derived cells (C), trachea-derived cells (D), stomach-derived cells (E), small intestine-derived cells (F), rectum-derived cells (G), kidney-derived cells, bladder-derived cells (H), are illustrated in UMAP plots. [file Image_7.tif]

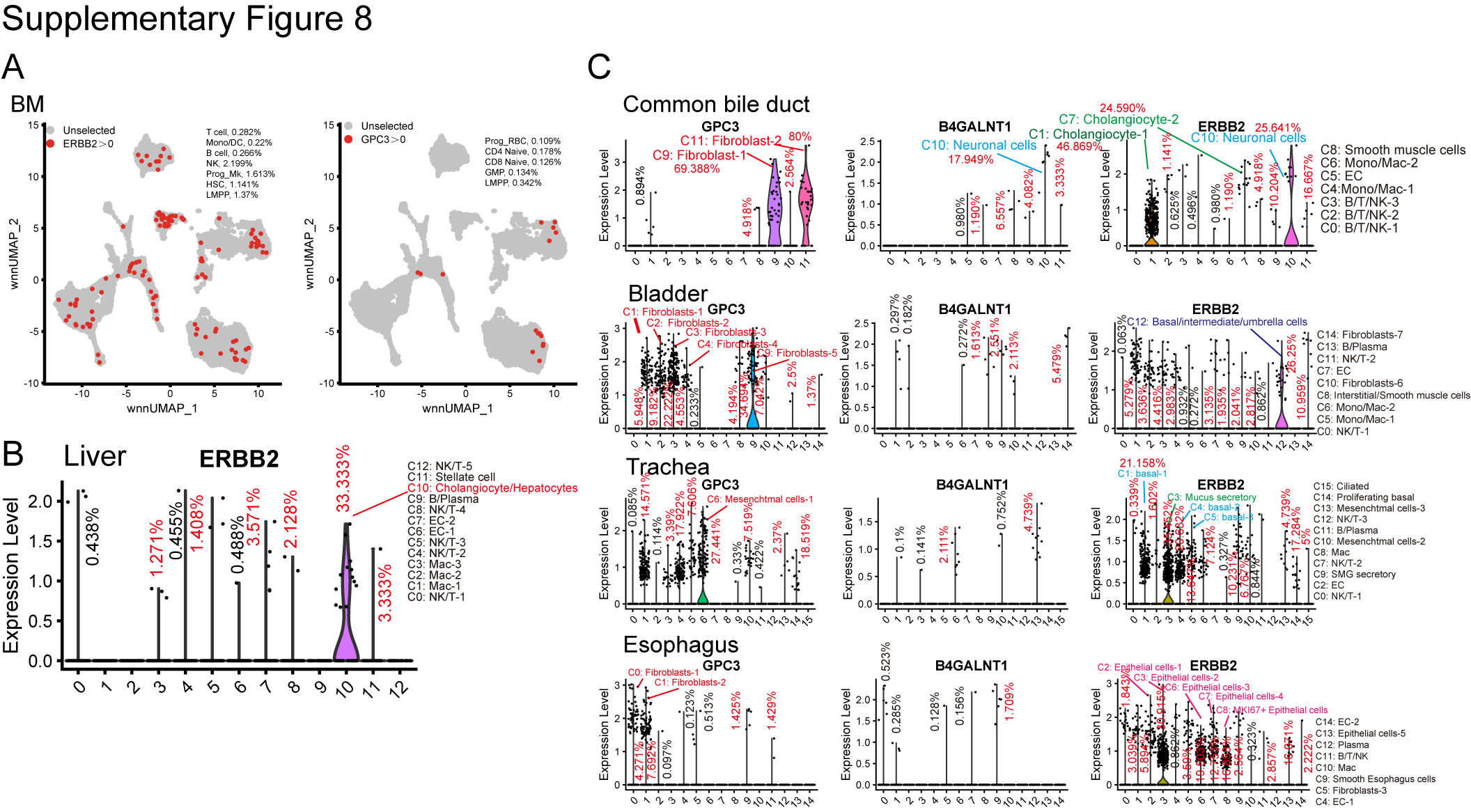

Supplement: Supplementary Figure 8 — Expression levels of solid tumor antigens (GPC3, B4GALNT1, and ERBB2) in human normal tissues and organs. (A) GPC3 and ERBB2-expressing proportions (expression value > 0) of BM-derived cells. (B) Violin plot shows the expression level of ERBB2 in liver-derived clusters. (C) Violin plots indicate the expression levels of GPC3, B4GALNT1, and ERBB2 in common bile duct-derived clusters, bladder-derived clusters, trachea-derived clusters, and esophagus-derived clusters. [file Image_8.tif]

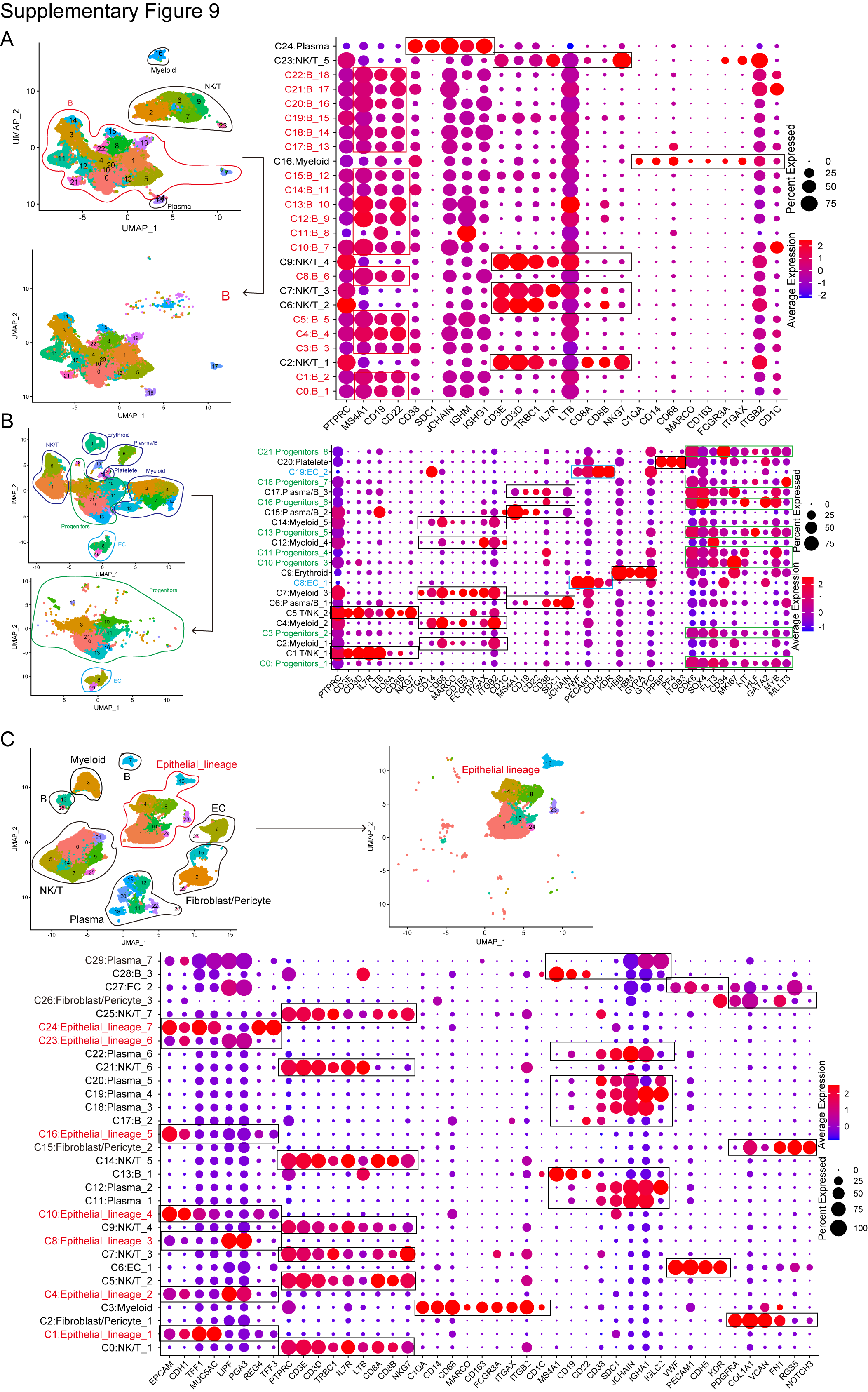

Supplement: Supplementary Figure 9 — (A) UMAP projection of lymphoma- and rLN-derived cells, and dot plot showing the expression levels of representative genes for different cell types. (B) UMAP projection of AML- and healthy donor-derived BM cells, and dot plot showing the expression levels of representative genes for different cell types. (C) UMAP projection of single cells of normal primary gastric tissue and gastric cancer, and dot plot showing the expression levels of representative genes for different cell types. [file Image_9.tif]

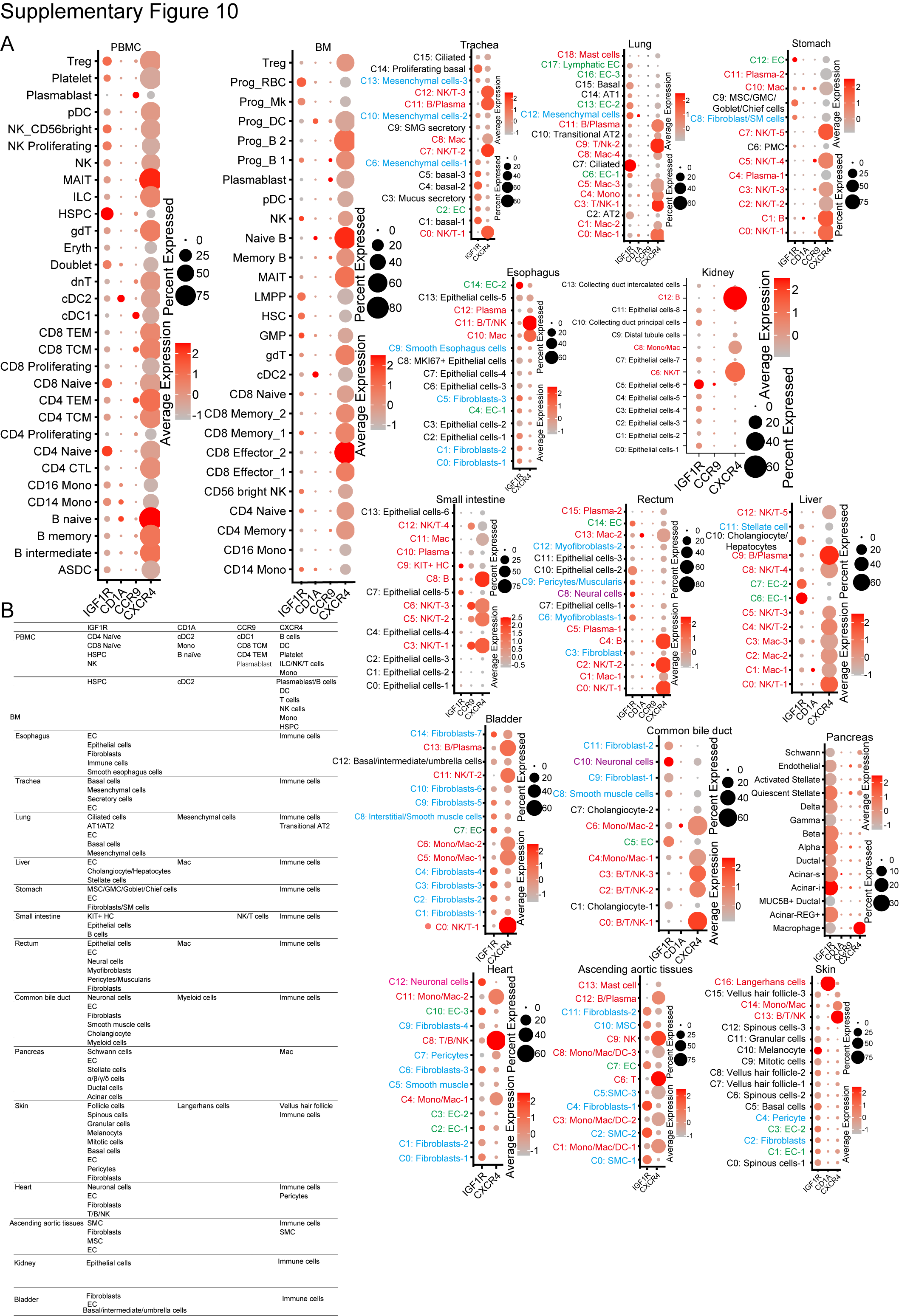

Supplement: Supplementary Figure 10 — (A) Dot plots show the expression levels of target antigens (IGF1R, CD1A, CCR9, and CXCR4) in PBMCs, BM, Trachea, lung, stomach, esophagus, kidney, small intestine, rectum, liver, bladder, common bile duct, pancreas, heart, ascending aortic tissue, and skin. (B) Summarization of high IGF1R/CD1A/CCR9/CXCR4-expressing subpopulations in various tissues and organs. [file Image_10.tif]
